# Nucleic-Acid-Binding Chromophores as Efficient Indicators of Aptamer-Target Interactions

**DOI:** 10.1155/2012/247280

**Published:** 2012-10-10

**Authors:** Kwabena Sarpong, Bhaskar Datta

**Affiliations:** ^1^Department of Chemistry, Missouri State University, 901 South National Avenue, Springfield, MO 65897, USA; ^2^Department of Chemistry, Indian Institute of Technology Gandhinagar, VGEC Complex, Chandkheda, Ahmedabad 382424, India

## Abstract

The binding affinity and specificity of nucleic acid aptamers have made them valuable candidates for use as sensors in diagnostic applications. In particular, chromophore-functionalized aptamers offer a relatively simple format for detection and quantification of target molecules. We describe the use of nucleic-acid-staining reagents as an effective tool for detecting and signaling aptamer-target interactions. Aptamers varying in size and structure and targeting a range of molecules have been used in conjunction with commercially available chromophores to indicate and quantify the presence of cognate targets with high sensitivity and selectivity. Our assay precludes the covalent modification of nucleic acids and relies on the differential fluorescence signal of chromophores when complexed with aptamers with or without their cognate target. We also evaluate factors that are critical for the stability of the complex between the aptamer and chromophore in presence or absence of target molecules. Our results indicate the possibility of controlling those factors to enhance the sensitivity of target detection by the aptamers used in such assays.

## 1. Introduction

Over the past two decades, the principles underlying nucleic acid structure and the polymeric characteristics of oligonucleotides have been exploited for creation of special ligand sequences that bind specific target molecules with high affinity and specificity [1, 2]. These special sequences or aptamers are discovered from combinatorial oligonucleotide libraries by iterative selection and amplification processes. The rationale for an iterative selection process is explained by the principles of systematic evolution of ligands by exponential enrichment (SELEX) [2]. SELEX has allowed the discovery of aptamers for a plethora of molecular targets including small molecules and proteins [3]. By virtue of their high affinity and specificity aptamers have emerged as a class of molecules that rival antibodies especially in diagnostic and biosensing applications [4]. In particular, aptamers address several shortcomings of antibodies such as the ability to function in nonphysiological buffers and temperatures and the ability to identify reporter molecules such as fluorescein at precise locations. In this regard, aptamer-based optical sensors are extremely attractive and have been the object of extensive research [5–8]. Molecular beacon-inspired optical assays require functionalization of the 5′- and/or 3′-termini of an aptamer with a fluorophore and a quencher. In the presence of or upon binding to the target, conformational changes in the aptamer manifest themselves as changes in fluorescence [9]. Single-or dual-labeled aptamers can be used to detect target binding by monitoring the target-induced FRET or anisotropy changes [10]. These methods provide real-time information on aptamer-ligand interactions compared to techniques such as autoradiography and electrophoresis. While the experimental design in such an approach may appear straight forward, it presupposes adequate knowledge of the structure and various conformations accessible to the aptamer in the presence and absence of target molecules. Labeling of aptamers with reporter molecules usually requires extensive optimization as small changes in the aptamer sequence and/or conformation may lead to unwanted changes in affinity and specificity [11, 12].

 A general method that allows direct transduction of the recognition event into a change in optical spectra would thus be extremely valuable [13]. In particular, the association of DNA-binding chromophores with nucleic acid aptamers could be affected in varying degrees by the formation of aptamer-analyte complexes. Previous approaches in this realm have generally been in a dye-displacement format where an RNA or DNA aptamer binds to a chromophore, resulting in a fluorescent complex [7, 14]. Presence of the target in solution results in the displacement of dye from the complex and reduction in fluorescence. While a number of chromophores have been tested in this format, kinetic barriers may prevent efficient displacement of the dyes from the complex. Also, previous reports have primarily focused on only single chromophores for monitoring specific aptamer-target interactions without any comparison of different chromophores across a range of aptamers that significantly differ in secondary structures. In this work, we use a number of commercially available nucleic-acid-binding dyes for monitoring aptamer-target interactions [15, 16]. We monitor the formation of the fluorescent complex by nucleic acid and chromophore in the presence and absence of cognate target. Our study reveals that the chromophores SYBR Green and thiazole orange (TO) can be used across RNA and DNA aptamers, albeit with different efficiencies in each specific case. We demonstrate the potential of rationally designed aptamer-chromophore pairs for sensitive and selective detection of a wide variety of targets.

## 2. Experimental and Methods

### 2.1. Aptamers

The structures of the aptamers employed for our current work have been previously published [17, 18]. Both the thrombin and ATP DNA aptamers are guanine-rich and fold into quadruplex helical structures, forming a pocket which allows for the binding of their target molecules [19], while the theophylline aptamer is an RNA oligonucleotide that folds into a complex stem-loop structure [20]. The sequences of the aptamers used in this work are shown in Table 1.

### 2.2. Dyes/Labels

Five different dyes that are known for binding nucleic acids were utilized for our fluorescence studies. These are shown in Figure 2. Stock solution of ethidium bromide was obtained from Fisher Bioreagents (Fair Lawn, NJ), diluted with double deionized water to different standards to ascertain the optimum concentration needed for fluorescence studies. Hoechst dye 33258 was purchased from Fischer Scientific (Pittsburgh, PA) and a 1 mM solution prepared in methanol. YOYO-1 was purchased from Invitrogen (Eugene, OR) and diluted to a 40 *μ*M solution in DMSO. Thiazole orange (TO) was obtained from AnaSpec Inc. (San Jose, CA), dissolved in DMSO and a 1 mM solution prepared. Stock solution of SYBR Green was purchased from Invitrogen (Eugene, OR) and diluted with DMSO to obtain a 10x concentrated solution. All the dyes exhibit high quantum yields of fluorescence upon binding nucleic acids.

### 2.3. DNA/RNA Synthesis

Oligonucleotides were either purchased from Integrated DNA Technologies (Coralville, IA) or synthesized on an Applied Biosystems 392 DNA/RNA Synthesizer using standard phosphoramidite chemistry, and reagents from Glen Research (Sterling, VA). Oligonucleotides were ammonia deprotected at 55°C overnight and purified using low-melting gel electrophoresis or by using reverse-phase cartridges on oligonucleotides synthesized with the hydrophobic dimethoxytrityl (DMT) group. Extinction coefficients of the synthesized oligonucleotides were calculated by the nearest neighbor method [22]. Absorbance values of purified sequences were obtained at 25°C and pH 7.0 phosphate buffer and used to calculate the working concentrations of our DNA/RNA sequences.

### 2.4. Target Molecules

Theophylline was purchased from Acros Organics (Morris Plains, NJ). ATP was obtained from Fisher Bioreagents (Fair Lawn, NJ) and thrombin was purchased from Fischer Scientific (Pittsburgh, PA). 100 *μ*M solutions of these were prepared in sterile water except for human *α*-thrombin which was prepared in a 50 mM sodium citrate/0.2 M NaCl/0.1% PEG-8000/pH 6.5 solution according to the vendor's instructions. Appropriate final concentrations of the target molecules were obtained by dilution of the above stock solutions.

### 2.5. Sample Preparation

The current study explores the use of nucleic-acid-binding chromophores in a “staining” rather than a “dye-displacement” format. Samples were prepared as per the following procedure.

#### 2.5.1. Aptamer Annealing

100 nM ATP and thrombin aptamers were prepared in 20 mM Tris-Cl buffer pH 7.5 with 300 mM NaCl, 5 mM MgCl_2_, and 10 mM KCl whereas 100 nM theophylline aptamer was prepared in a 20 mM Tris-Cl buffer pH 7.5, 140 mM NaCl, 5 mM KCl, and 5 mM MgCl_2_ with the addition of sterile water to make a 1 mL solution. These were annealed at 85–90°C for at least 5 minutes followed by gradual cooling to room temperature.

#### 2.5.2. Addition of Cognate Target

Aptamer samples were prepared in duplicate following the procedure described above. For each aptamer, one sample was incubated with its cognate target while the other was not. The volume of addition in each case was designed not to exceed 10 *μ*L, thereby contributing to almost insignificant changes in total concentrations of aptamer and target. Aptamers were allowed to incubate with their target for 30 minutes in an incubator at 25°C. Longer incubation times (1–12 hours) were found to yield similar results. All samples were prepared in triplicate for fluorescence studies.

### 2.6. Fluorescence Studies

Chromophores were added into the samples containing aptamers with and without targets following incubation. Chromophore concentrations were found to be optimal at 250 nM (for probe and target concentrations of 100 nM each). Emission spectra were recorded at 25°C on a Shimadzu RF-5301 PC spectrophotometer by exciting dyes at their respective excitation wavelengths. Excitation and emission slit widths were maintained at 5 nm throughout all experiments. The following excitation wavelengths were used: ethidium bromide, 340 nm; Hoechst dye 33258, 320 nm; thiazole orange, 510 nm; SYBR Green, 497 nm; YOYO-1, 488 nm. Emission intensities at characteristic emission maxima for each chromophore were used for calculating the percentage decrease in fluorescence upon an aptamer-binding cognate target using the following formula:

(1) $$\begin{array}{c} \%\text{Decrease}\;\text{in}\;\text{fluorescence}=\frac{F_{o}-F_{i}}{F_{o}}\times 100, \end{array}$$

where *F*_*o*_ is fluorescence of dye + aptamer, and *F*_*i*_ is fluorescence of dye + aptamer-target.

Experiments that are performed to measure *F*_*o*_ and *F*_*i*_ have the same amounts of dye, aptamer,and target. Based on the percentage decreases observed throughout this study, a drop in fluorescence of ≤10% is considered to be within the range of experimental error mainly due to variations in the amounts of dye added. Experiments were performed in triplicate and the errors shown are standard deviation from the mean.

## 3. Results and Discussion

### 3.1. Comparison of Chromophores as Indicators of Aptamer-Target Interactions

**Figure 1:**
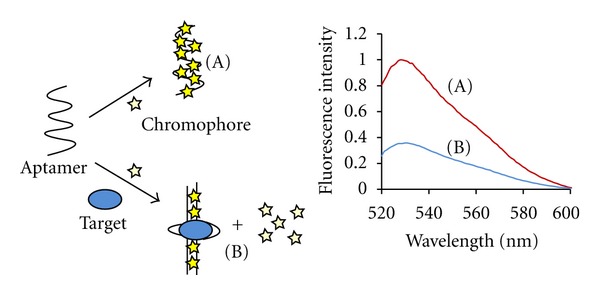
Strategy for using nucleic acid binding chromophores as indicators of aptamer-target interactions. The plot on the right shows fluorescence spectra of Thiazole Orange (TO) in complex with the theophylline aptamer (A) in the absence and (B) presence of target.

**Figure 2:**
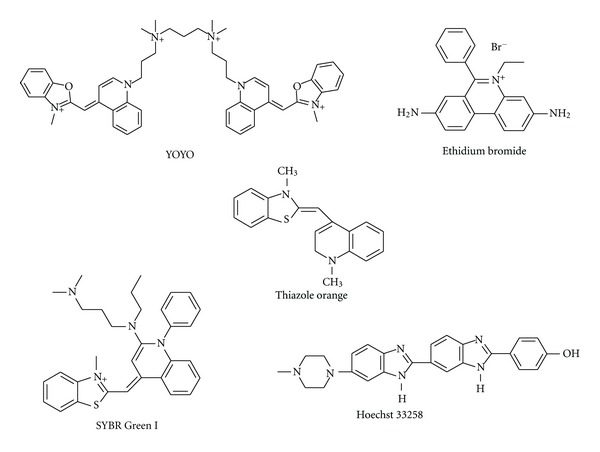
Nucleic-acid-binding chromophores used in current study.

**Figure 3:**
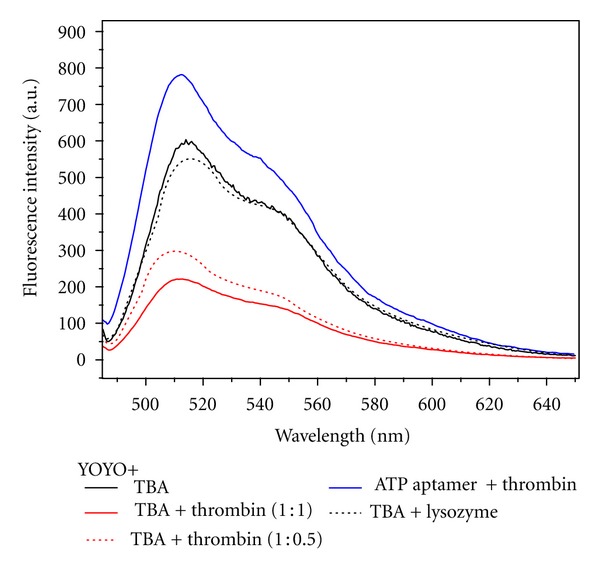
Comparison of fluorescence emission spectrum of YOYO in presence of TBA, TBA + thrombin (in two ratios), ATP aptamer and TBA + lysozyme. Samples contain 25 nM of YOYO and 10 nM of aptamers. Amount of thrombin used was 10 nM and 5 nM corresponding to 1 : 1 and 1 : 0.5 with respect to TBA.

**Figure 4:**
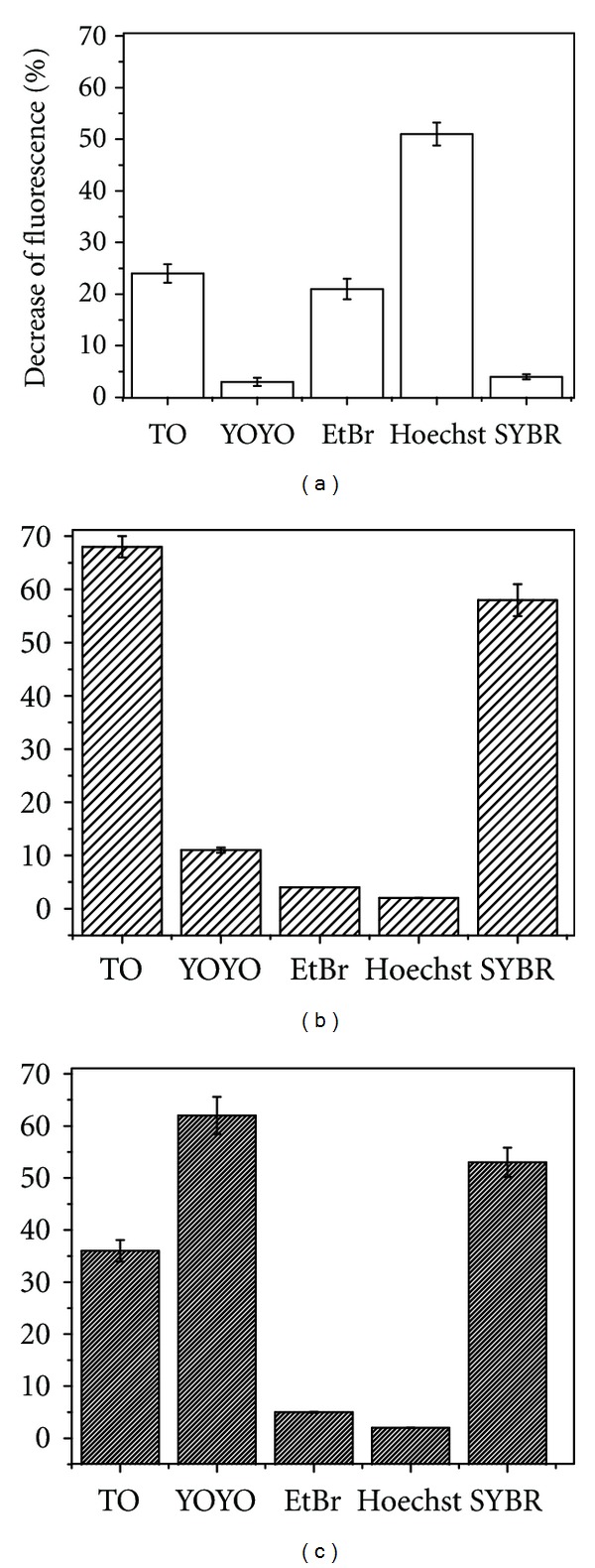
Comparison of fluorescence signaling by various chromophores on the (a) ATP-, (b) theophylline- and (c) thrombin-binding aptamers. The % decrease of fluorescence (%) is calculated from the difference in fluorescence intensities of the chromophores (25 nM) in presence of 10 nM aptamer with and without 10 nM target. Data presented in plot is the mean of three measurements and error bars indicate standard deviations.

**Figure 5:**
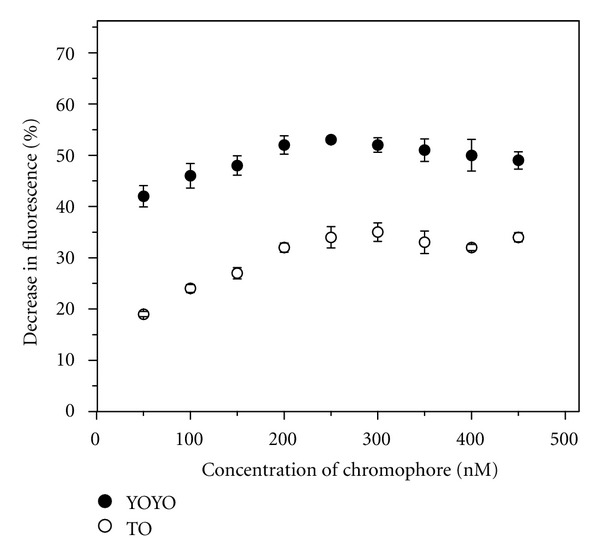
Variation of fluorescence signaling by YOYO and TO with change in their concentrations in presence of 10 nM each of TBA and thrombin.

The signaling strategy used in this work is depicted in Figure 1. Briefly, the chromophores being used in this assay are almost nonfluorescent when free in solution but exhibit a strong increase in fluorescence quantum yield when bound to single or double-stranded DNA. Structural perturbations in the nucleic acid aptamers upon binding cognate targets are expected to generate measurable changes in the fluorescence binding behavior of chromophores. The success of the signaling strategy thus not only depends on the degree to which aptamers undergo conformational changes upon target binding but also on the identity of the chromophore used that may or may not be sensitive to those changes. Structures of the chromophores used in the current work are shown in Figure 2. In order to explore the general applicability of the strategy shown in Figure 1, we also chose to study a number of different aptamer-target pairs (shown in Table 1) to account for differences in type of nucleic acid (RNA or DNA), binding affinities for targets (shown in Table 1), type of secondary structures (hairpins, quadruplexes, or both), and binding motifs applicable to the chromophore (intercalation, minor groove binding). The behavior of YOYO as per the signaling strategy described above is shown in Figure 3. Binding of thrombin by TBA leads to a significant drop in fluorescence emission of YOYO thereby indicating sensitivity of the dye to conformational changes in the aptamer. The specificity of TBA and of our approach is evident from the lack of significant decrease in fluorescence of YOYO for the TBA-lysozyme and ATP aptamer-thrombin controls. In fact the fluorescence of YOYO in presence of ATP aptamer is very similar in absence of target ATP (data not shown). The larger absolute fluorescence observed is possibly due to the availability of larger number of binding sites in the ATP aptamer compared to TBA. The ATP aptamer chosen for this work possesses an ATAT tract which is known to assist in Hoechst 33258 binding [23]. Notably, comparing the decrease in fluorescence across chromophores (see Figure 4(a) and Table S1 in Supplementary Material available online at doi:10.1155/2012/247280), the change in the Hoechst 33258 fluorescence is the greatest. The percentage changes in Figure 4 were calculated by comparing the fluorescence of each chromophore complexed to ATP aptamer with and without the target. Experiments using a range of chromophore concentrations within an order of magnitude were found to yield very similar results. A small percentage change (≤10%) is possibly indicative of an aptamer-chromophore complex that is not significantly perturbed in presence of the target. While both the ATP aptamer and the thrombin-binding aptamer (TBA) possess quadruplex structural elements, the latter does not have a duplex tract. Comparing the decrease in fluorescence across chromophores, it is thus not surprising that the Hoechst 33258 dye displays negligible change (see Figure 4(c)). The intercalating dyes, thiazole orange (TO) and SYBR Green, exhibit weak-to-moderate fluorescence decreases for TBA in the presence of thrombin target. YOYO exhibits the greatest decrease in fluorescence for TBA in presence of thrombin. The relatively short sequence of TBA and corresponding compact structure offer fewer sites for chromophore binding compared to the ATP aptamer. This is responsible for the attenuation of fluorescence change across chromophore concentrations. As shown in Figure 5, there is little variation in the fluorescence change of YOYO even when the chromophore concentration is varied over an order of magnitude. Interestingly ethidium bromide only exhibits poor fluorescence changes in the presence versus absence of thrombin, even though its binding mode is similar to that of YOYO and TO. This is possibly an indication of the weaker binding affinity of ethidium bromide for nucleic acids compared to the other staining reagents. The substantial drop in fluorescence of YOYO (~60%) for TBA in the presence of thrombin may be due to the G-quartet motif of TBA providing a secure binding pocket for YOYO, which is then perturbed upon target (thrombin) binding. Finally, we tested the intercalating dyes, TO, SYBR Green, and YOYO, on an RNA theophylline aptamer (see Figure 4(b)). Both SYBR green and TO display a significant decrease in fluorescence for the aptamer in presence of theophylline target. The magnitude of fluorescence changes is greater for the theophylline aptamer (~68%) compared to ATP and thrombin aptamer possibly due to higher binding affinity of the dyes for RNA molecules and greater conformational changes in aptamer upon target binding.

### 3.2. Sensitivity and Selectivity of Chromophores Used for Monitoring Aptamer-Target Interactions

**Figure 6:**
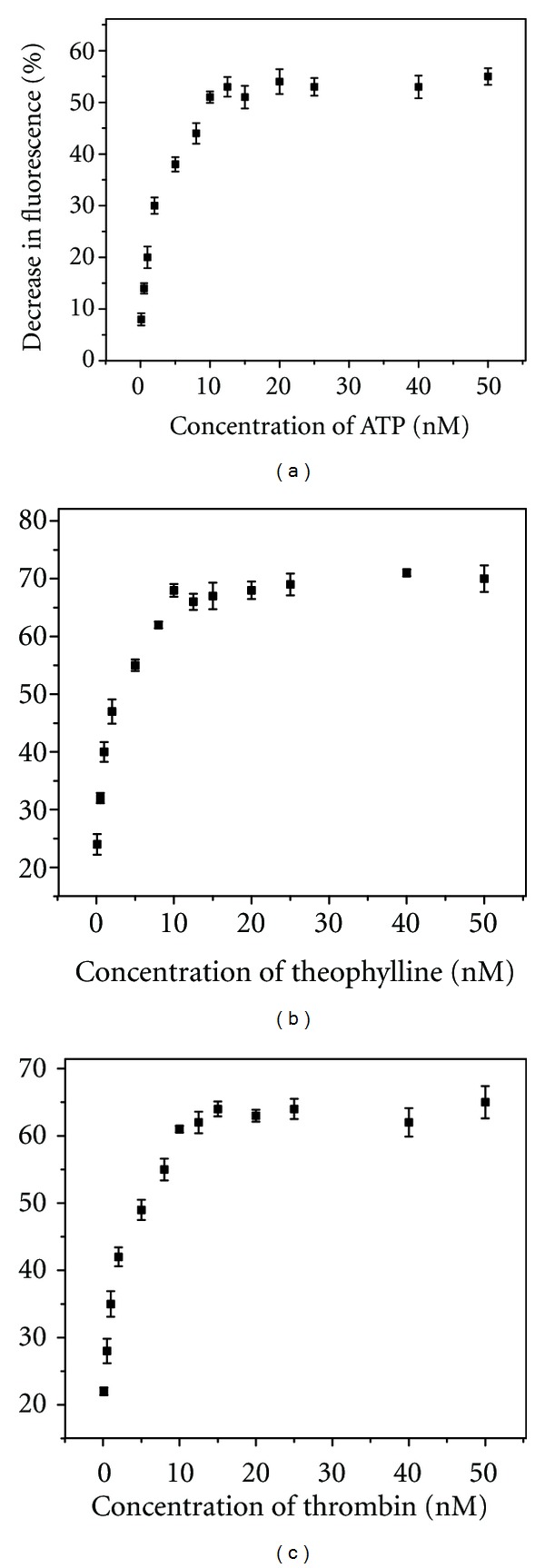
Fluorescence signaling by chromophores in presence of aptamers exposed to variable amounts of cognate target. Chromophores used for (a) ATP, (b) theophylline, and (c) thrombin were, respectively, Hoechst 33258, TO, and SYBR Green I. The % decrease in fluorescence is calculated from the difference in fluorescence intensities of 25 nM chromophores in presence of 10 nM aptamer and variable amounts of cognate target.

**Figure 7:**
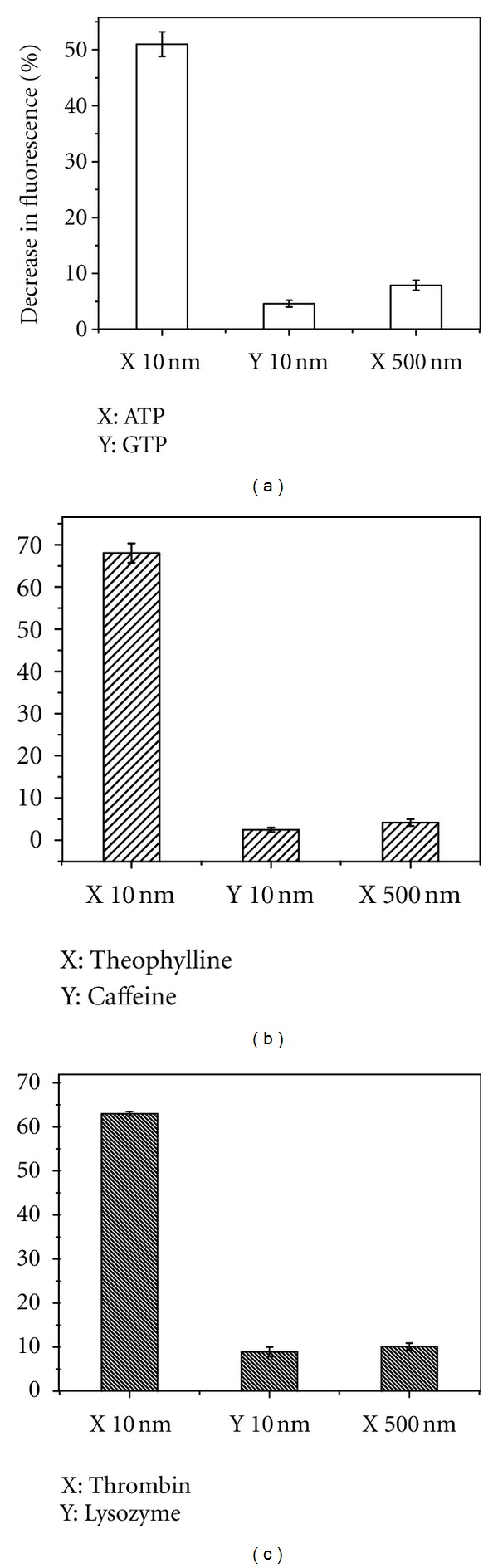
Specificity of signaling strategy towards aptamer and cognate target. Chromophores used for the (a) ATP, (b) theophylline, and (c) thrombin aptamers, respectively, were Hoechst 33258, TO, and YOYO. Change of fluorescence is the difference in fluorescence intensities of chromophores (25 nM) with 10 nM aptamers and presence or absence of indicated amounts of target.

We tested the sensitivity of our approach by varying the amount of cognate target for each of the three aptamers. Not surprisingly the aptamer-dye pairs that provided the best fluorescence response were also the most sensitive towards target concentration. Notably, the thrombin aptamer in conjunction with YOYO was able to detect 0.1 nM of thrombin, albeit with only ~20% decrease in fluorescence of the chromophore (see Figure 6). Similarly, 1 nM of theophylline and 2 nM of ATP induced a measurable change in fluorescence intensity of TO and Hoechst 33258, respectively. These detection limits (~0.1–2 nM) are somewhat comparable with the use of chromophores that are covalently coupled to aptamer probes [9, 24]. Finally we tested our approach for selectivity of an aptamer towards its cognate target. In this regard, the theophylline aptamer has been shown to be selective for theophylline over caffeine notwithstanding structural similarity between the two molecules. As shown in Figure 7, the theophylline aptamer in conjunction with TO is insensitive to caffeine concentration with even a huge excess of caffeine failing to elicit a fluorescence change of less than 5%. Similarly, the ATP aptamer in conjunction with Hoechst 33258 when tested against the structurally analogous GTP target elicits substantially smaller fluorescence changes (~5%). Finally, the selectivity of TBA is evident from the lack of change in fluorescence of YOYO in presence of lysozyme as compared to thrombin. These results demonstrate the selectivity of our approach using nucleic acid staining dyes for detecting aptamer-target interactions.

### 3.3. Factors Affecting the Signaling Strategy

From our study of both DNA and RNA aptamers using numerous commercially available staining agents some factors have been identified as being key to the success of exploiting the signaling strategy. First, the presence of distinctive binding sites for chromophores within the aptamers would likely help the signaling strategy. While this feature may be difficult to incorporate for intercalating chromophores, it is possible to develop aptamers that possess specific tracts for groove-binding dyes. Second, chromophores that exhibit stronger binding affinity for a specific nucleic acid aptamer are more likely to succeed at this signaling strategy. Relatively weaker binding chromophores would be less perturbed by aptamer-target interactions and would thus be less sensitive to analyte concentrations. Finally, the success of the strategy depends in large part on the affinity and specificity of the aptamer for its cognate target. The better performance of the thrombin- and theophylline-binding aptamers compared to the ATP aptamer can be attributed to the higher binding affinity of those aptamers for their corresponding targets. A higher binding affinity ensures aptamer-target complexation at low concentrations of target. More efficient formation of aptamer-target complexes (as a fraction of total amount of aptamer and target present) by the higher binding affinity aptamers would also allow for better signalling by chromophores. 

## 4. Conclusions

An aptamer-based method for detection of analytes has been developed which successfully avoids the more common chemical labeling and modification procedures. Covalent labeling of aptamers with reporter molecules including fluorophores usually lead to higher cost and unpredictable affinity of the aptamer for its target [25]. The reported method is thus a simple economical alternative to selective and sensitive detection of analytes. We have performed a comprehensive analysis of numerous chromophores across several aptamer-target pairs unlike previous reports that focused on single candidates of each [26–28]. One drawback of unlabeled nucleic acid aptamers for detecting physiological analytes is possible interference from residual nucleic acids from such samples. We are currently exploring these parameters further to develop efficient signaling chromophores. 

## Supplementary Material

UV-thermal melting plots of the aptamers used in this investigation and magnitude of fluorescence decrease of various chromophores used on the aptamer-target pairs are provided as supplementary information.

## Figures and Tables

**Table 1:** Sequences of aptamers used and their affinities for corresponding target.

Target	Sequence	Affinity (*K*_*d*_)	Expected secondary structure
ATP [17]	5′-ATAGCGGAGGAAGGATACCTGGGGGAGTATAT-3′	6 *μ*M	Quadruplex, stem-loop
Thrombin [18]	5′-GGTTGGTGTGGTTGG-3′	10 nM	Quadruplex
Theophylline [21]	5′-GGUGAUACCAGCAUCGUCUUGAUGCCCUUGGCAGCACC-3′	220 nM	Hairpin, stem-loop
