# Supplementary material for: Nucleic-Acid-Binding Chromophores as Efficient Indicators of Aptamer-Target Interactions
Source: J Nucleic Acids. 2012 Oct 10;2012:247280. doi: 10.1155/2012/247280 (PMC3474971; doi:10.1155/2012/247280)
Supplement: Supplementary file 1 — UV-thermal melting plots of the aptamers used in this investigation and magnitude of fluorescence decrease of various chromophores used on the aptamer-target pairs are provided as supplementary information. [file 247280.f1.pdf]

Nucleic Acid Binding Chromophores as Efficient Indicators of Aptamer-Target Interactions

Supplementary Information

Kwabena Sarpong and Bhaskar Datta[*,†]

Department of Chemistry, Missouri State University, 901 S. National Avenue, Springfield MO 65897.

* Corresponding author

* Bhaskar Datta, Department of Chemistry, Missouri State University, 901 S. National Avenue, Springfield MO 65897

†Current Address: Department of Chemistry, Indian Institute of Technology Gandhinagar, VGEC Complex, Chandkheda, Ahmedabad 382424 India

Telephone: 91-79-3245 9902; Fax: 91-79-2397 2622; E-mail: bdatta@iitgn.ac.in

Table S1. Percentage decrease in fluorescence of dyes used on various aptamer-target complexes

**Aptamer (Target)**	Thiazole Orange	YOYO	Ethidium Bromide	Hoechst 33258	SYBR Green I
ATP	24 ± 1.8	3 ± 0.8	21 ± 2	51 ± 2.2	4 ± 0.5
Theophylline	68 ± 1.1	11 ± 0.5	4 ± 1	2 ± 0.6	57 ± 2.3
Thrombin	36 ± 2.1	62 ± 0.5	5 ± 0.5	2 ± 0.1	53 ± 1.8

Percentage decreases were calculated as described in the experimental. Errors bars indicated are standard deviations from three separate measurements.

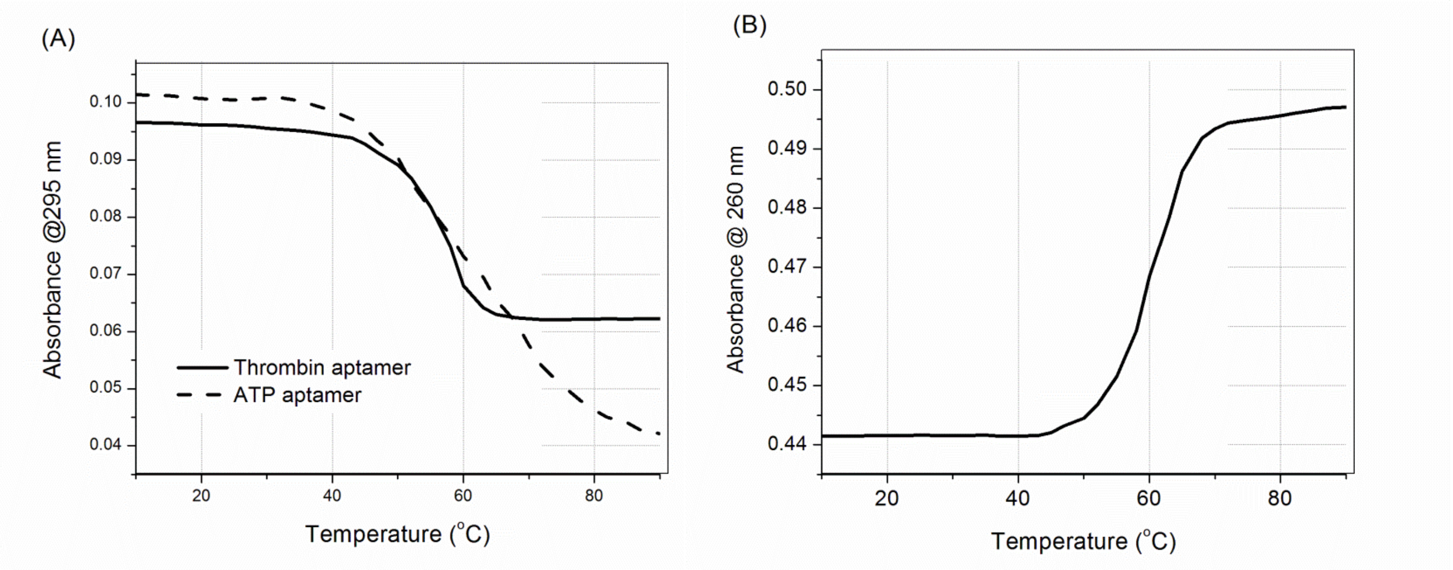

Figure S1. UV-thermal melting curves of (A) thrombin and ATP, and (B) theophylline aptamers. Experiments were performed on samples containing 2 μM of nucleic acid aptamers in identical conditions to those used for aptamer annealing. Samples were annealed first and dissociation profiles were measured at the rate of 1 ^{o}C/min on a Perkin Elmer Lambda 650 spectrophotometer equipped with a PTP 1+1 Peltier system temperature controller. For the thrombin and ATP aptamers, absorbance was measured at 295 nm, while the theophylline aptamer melting profile was measured at 260 nm. Melting temperatures were calculated by taking first derivatives of the melting curves and were as follows: Thrombin aptamer: 58 ^{o}C; ATP aptamer: 52 ^{o}C; and theophylline aptamer: 60 ^{o}C.
